# A Calcium/Cation Exchanger Participates in the Programmed Cell Death and *in vitro* Virulence of *Entamoeba histolytica*

**DOI:** 10.3389/fcimb.2018.00342

**Published:** 2018-10-01

**Authors:** Martha Valle-Solis, Jeni Bolaños, Esther Orozco, Miriam Huerta, Guillermina García-Rivera, Andrés Salas-Casas, Bibiana Chávez-Munguía, Mario A. Rodríguez

**Affiliations:** ^1^Departamento de Infectómica y Patogénesis Molecular, CINVESTAV-IPN, Mexico City, Mexico; ^2^Área Académica de Gerontología, Instituto de Ciencias de la Salud, Universidad Autónoma del Estado de Hidalgo, Pachuca, Mexico

**Keywords:** *Entamoeba histolytica*, calcium/cation exchanger, protein overexpression, programmed cell death, virulence

## Abstract

*Entamoeba histolytica* is the etiologic agent of human amoebiasis, disease that causes 40,000 to 100,000 deaths annually worldwide. The cytopathic activity as well as the growth and differentiation of this microorganism is dependent on both, extracellular and free cytoplasmic calcium. However, few is known about the proteins that regulate the calcium flux in this parasite. In many cells, the calcium extrusion from the cytosol is performed by plasma membrane Ca^2+^-ATPases and calcium/cation exchangers. The aim of this work was to identify a calcium/cation exchanger of *E. histolytica* and to analyze its possible role in some cellular processes triggered by calcium flux, such as the programmed cell death and *in vitro* virulence. By searching putative calcium/cation exchangers in the genome database of *E. histolyica* we identified a protein belonging to the CCX family (EhCCX). We generated a specific antibody against EhCCX, which showed that this protein was expressed in higher levels in *E. histolytica* than its orthologous in the non-pathogenic amoeba *E. dispar*. In addition, the expression of EhCCX was increased in trophozoites incubated with hydrogen peroxide. This *E. histolytica* exchanger was localized in the plasma membrane and in the membrane of some cytoplasmic vesicles. However, after 10 min of erythrophagocytosis, EhCCX was found predominantly in the plasma membrane of the trophozoites. On the other hand, the parasites that overexpress this exchanger contained higher cytosolic calcium levels than control, but the extrusion of calcium after the addition of hydrogen peroxide was more efficient in EhCCX-overexpressing trophozoites; consequently, the programmed cell death was retarded in these parasites. Interestingly, the overexpression of EhCCX increased the *in vitro* virulence of trophozoites. These results suggest that EhCCX plays important roles in the programmed cell death and in the *in vitro* virulence of *E. histolytica*.

## Introduction

The ion calcium (Ca^2+^) is a highly versatile secondary messenger that operates over a wide temporal range to regulate many different cellular processes (Berridge et al., 2003). Thus, the concentration of Ca^2+^ in cytosol and some organelles, such as the endoplasmic reticulum, the Golgi apparatus, and nucleus, is strongly regulated by channels, transporters and pumps, which dynamically adjust the Ca^2+^ amount in agreement with a specific physiological demand (Berridge et al., 2003). In many cells Ca^2+^ extrusion from the cytosol is performed by plasma membrane Ca^2+^-ATPases (PMCAs) and by Ca^2+^/cations exchangers (Berridge et al., 2003). PMCAs have high Ca^2+^ affinity, but low turnover rates, while exchangers have a lower Ca^2+^ affinity, but higher turnover rates (Blaustein and Lederer, 1999). Consequently, PMCAs are involved in the maintenance of the resting cytoplasmic concentration of Ca^2+^, whereas exchangers are important in the restoration of cytoplasmic concentration of Ca^2+^ after it has been elevated during signaling (Harper and Sage, 2016). Interestingly, exchangers also participate in the entry of Ca^2+^ to cytoplasm, since they can reverse its transport in response to changes in the concentration of the transported ions and membrane potential (Harper and Sage, 2016). The superfamily of calcium exchangers comprises five branches (Lytton, 2007; Emery et al., 2012): (i) H^+^/ Ca^2+^ exchangers (CAX), mainly found in plants; (ii) bacteria exchangers (YRGB); (iii) Na^+^/Ca^2+^ exchangers (NCX or SLC8); (iv) Na^+^/Ca^2+^ + K^+^ exchangers (NCKX or SLC24); and (v) Ca^2+^/cation exchangers (CCX), which contains one mammalian member, the Na^+^/Ca^2+^ or Li^+^ exchanger (NCLX) that is also named as NCKX6.

*Entamoeba histolytica* is the etiological agent of human amoebiasis, a disease that produces 40,000 to 100,000 deaths per year worldwide (Stanley, 2003). The cytolytic activity of this parasite is dependent on both, extracellular and free cytoplasmic Ca^2+^ (Ravdin et al., 1982, 1985). In addition, Ca^2+^ flux participates in the adherence of trophozoites to fibronectin (Carbajal et al., 1996), as well as in growth and differentiation of *E. histolytica* and *E. invadens* (Makioka et al., 2001, 2002; Martínez-Higuera et al., 2015). However, little is known about the proteins that regulate the Ca^2+^ flux in this parasite. Our previous studies showed that *E. histolytica* contains at least five Ca^2+^-ATPases: three related to PMCAs, one to Sarco/Endoplasmic reticulum Ca^2+^-ATPases (SERCA), and another to Secretory Pathway Ca^2+^-ATPases (SPCA) (Martinez-Higuera et al., 2013; Rodríguez et al., 2018). Indeed, we detected the SPCA-related pump in the Golgi apparatus of *E. histolytica* (Rodríguez et al., 2018) and the SERCA-related pump in the endoplasmic reticulum of *E. histolytica* and *E. invadens* (Martinez-Higuera et al., 2013; Martínez-Higuera et al., 2015). In addition, we demonstrated that specific inhibitors of SERCA affected the encystation of *E. invadens* (Martínez-Higuera et al., 2015), suggesting that calcium flux through SERCA is involved in the development of *Entamoeba* sp. However, other proteins involved in the Ca^2+^ movement, such as channels or exchangers, and their role in the *Enatamoeba* biology have not been described.

In this work, we identified a calcium/cation exchanger of *E. histolytica* related to members of the CCX family (EhCCX). This exchanger was higher expressed in *E. histolytica* that its orthologous in the non-pathogenic amoeba *E. dispar*. EhCCX expression was increased in trophozoites incubated with hydrogen peroxide. The exchanger was located in the plasma membrane of trophozoites and in the membrane of some cytoplasmic vesicles, but after 10 min of erythrophagocytosis, EhCCX was mainly detected in the plasma membrane. On the other hand, the overexpression of EhCCX augmented the cytosolic calcium levels under basal conditions, but the calcium extrusion after the addition of hydrogen peroxide was more efficient. In addition, the overexpression of EhCCX retarded the programmed cell death and increased the *in vitro* virulence. These results suggest that the Ca^2+^ flux through EhCCX plays an important role in the programmed cell death and the virulence of *E. histolytica*.

## Materials and methods

### *Entamoeba* cultures

Trophozoites of *E. histolytica* clone A, strain HM1:IMSS (Orozco et al., 1983) were axenically cultured in TYI-S-33 medium (Diamond et al., 1978), whereas trophozoites of *E. dispar* (strain SAW 760) were axenically cultured in YI-S medium (Diamond et al., 1995). Cells were harvested during the logarithmic growth phase as previously described (Diamond et al., 1978).

### Identification and *in silico* characterization of a calcium/cation exchanger of *E. histolytica*

To identify possible calcium/cation exchangers in *Entamoeba* spp., a BLAST search was performed on the databases of the Amoeba Genomics Resource (http://amoebaDb.org/amoeba/) using as probes the α1 and α2 repeats of human calcium/sodium exchangers (NCX, NCKX, and NCLX). These motifs are characteristics of these transporters and participate in the ions transport (Philipson and Nicoll, 2000). Then, the retrieved protein of *E. histolytica* was characterized *in silico* using the software deposited in the Expasy Bioinformatics Resource Portal (http://expasy.org) and in the NCBI Home Page (http://www.ncbi.nlm.nih.gov). The amino acid sequence of this protein was compared, by ClustalW, with sequences of proteins belonging to the different families of calcium exchangers (CAX, YRGB, NCX, NCKX, and CCX). Then, a phylogenetic analysis was performed using the Unweighted Pair Group Method with Arithmetic Mean (UPGMA) employing the MEGA 5.05 software package (Tamura et al., 2011). Bootstrapping was performed for 1000 replicates.

The 3D molecular model was built with the I-TASSER server (http://zhanglab.ccmb.med.umich.edu/I-TASSER) (Zhang, 2008) and with the Raptor X Structure Prediction (http://raptorx.uchicago.edu) using as a template the crystalized structure of the *Methanococcus jannaschii* NCX (Protein Data Bank: 3V5S) (Liao et al., 2012).

### PCR and RT-PCR

The genomic DNA of *E. histolytica* was obtained with the Wizard Genomic DNA purification kit (Promega), following the manufacturer's recommendations. Total RNA was isolated using the Trizol reagent (Invitrogen), following the manufacturer's recommendations, and cDNA was synthesized using an oligo dT primer and the Superscrip II reverse transcriptase (Invitrogen). Amplifications of the *Ehccx* full-length gene was performed in a select Cycler (Select BioProducts) using 200 ng of DNA or cDNA and specific primers situated at the 5′- and 3′-ends of the gene and containing the *BamHI* and *KpnI* restriction sites, respectively (forward, 5′- CCCCGGTACCATGAAACAGATGAATAAAATTTATATTATATTA-3′; reverse, 5′-CCCCGGATCCTTAACCAAACAGTTTAAAAACGTTAA-3′). The assays were performed in a 50 μl volume reaction containing 1 μM of each primer, dNTPs 1.5 mM, MgCl_2_ 2 mM, and 1 U of the high-fidelity enzyme KAPA HiFi DNA polymerase (KAPABIOSYSTEMS). Amplification cycles comprised: (i) 1 min of denaturing step at 94°C; (ii) 35 cycles of 1 min of denaturing step at 94°C, 1 min of annealing step at 55°C; and 3 min of elongation step at 72°C; and (iii) 10 min of elongation step at 72°C. The amplified products were analyzed by electrophoresis in 1% agarose gels.

### Production of the α-EhCCX antibody and western blot

To obtain antigenic peptides of the predicted calcium/cation exchanger of *E. histolytica* (EhCCX), its amino acid sequence was analyzed by the ABCpred program (http://www.imtech.res.in/raghava/abcpred/). Peptides with higher scores were used as probes in BLAST searches on the *E. histolytica* genome database; then, a specific peptide at position 216-229 (ISEQLDSENKTKLI) was synthesized (GL Biochem) linked to the KLH (Keyhole limpet Hemocyanin) tag to increase its immunogenicity. Next, Wistar rats were intradermally immunized three times, in an interval of 2 weeks, with 100 μg of the synthetic polypeptide resuspended in Titermax Gold adjuvant (1:1) (Sigma). All animals used in this study were handled in accordance with the guidelines of the Institutional Animal Care and Use Committee. Our institution fulfills all the technical specifications for the production, care and use of laboratory animals and it is certified by national law (NOM-062-ZOO-1999).

For Western blot assays, total extracts of *E. histolytica* or *E. dispar* trophozoites obtained in the presence of protease inhibitors (Complete Mini, Roche-Mannheim) were separated by 10% SDS-PAGE, transferred to nitrocellulose membranes and probed with the α-EhCCX antibody (1: 3,000). Then membranes were incubated with a peroxidase-coupled secondary antibody (1: 10,000) (ZYMED) and finally, the reaction was developed by chemiluminescence (ECL Plus GE-Healthcare). To compare the expression level of EhCCX and EdCCX or to analyze the expression level of EhCCX in trophozoites under different conditions (in the presence of H_2_0_2_ 1 mM for 10 min, or incubated at 42°C for 30 and 60 min) we performed Western blot assays using the α-EhCCX antibody. Then, membranes were exposed to an α-actin antibody (1: 20,000) (kindly provided by Dr. Jose Manuel Hernandez-Hernandez at CINVESTAV-IPN, Mexico), used as an internal control of loading. The band detected by the α-EhCCX antibody was analyzed by scanning densitometry and the data were normalized to the actin content according to the reactivity of the α-actin antibody. For semi-quantitative comparisons, the protein level in *E. histolytica* trophozoites under basal conditions was arbitrary taken as 1.

### Immunofluorescence and confocal microscopy

Trophozoites grown on coverslides were fixed and permeabilized with methanol for 10 min and non-specific binding sites were blocked with 10% FBS in phosphate buffered saline (PBS). Then, cells were incubated for 1 h at 37°C with the rat antibody against EhCCX (1:50 dilution). After several washes with PBS, samples were incubated with an Alexa 555-conjugated secondary antibody (Zymed) (1:400). Finally, the nuclei were stained with 4',6-diamidino-2-Phenylindole (DAPI) and samples were observed through a confocal microscope (Carl Zeiss LSM 700). Observations were performed in ~20 optical sections from the top to the bottom of each sample. For the immunolocalization of EhCCX during phagocytosis, before performing the immunofluorescence, trophozoites were incubated with fresh human red blood cells (RBCs) from healthy donors (1:25 ratio) for 5, 10, and 15 min at 37°C. As a control, cells were incubated in the presence of EGTA 0.3 mM.

For co-localization assays, after incubation with α-EhCCX, the samples were incubated with rabbit antibodies against EhSERCA (Martinez-Higuera et al., 2013) (dilution 1:50), EhRabB (Rodriguez et al., 2000) (dilution 1:25), or EhNPC-1 (Bolaños et al., 2016) (dilution 1:100); or with mouse antibodies against the Gal/GalNac lectin (Petri et al., 1989) (dilution 1:50). Subsequently, the samples were incubated with anti-rat IgGs labeled with Alexa 555, and with anti-rabbit IgGs labeled with Alexa 488, or anti-mouse IgGs labeled with FITC (Zymed; 1:100) as appropriate. For detection of the Golgi apparatus, trophozoites were incubated with 5 μM of NBD C_6_-Ceramide (Thermo Fisher Scientific) for 90 min at 37°C. To evaluate the co-localization between molecules, Pearson coefficients were obtained from at least 15 confocal independent images (laser sections: 0.5 μm) using the ImageJ 1.45v software and the JACoP plugin.

### Immunoelectron microscopy

Immunoelectron microscopy assays were performed as described (Segovia-Gamboa et al., 2011). *E. histolytica* trophozoites were fixed in 4% paraformaldehyde and 0.1% glutaraldehyde in serum-free DMEM for 1 h at room temperature. Samples were embedded in LR white resin (London Resin Co) and polymerized under UV at 4°C for 48 h. Then, thin sections (60 nm) were mounted on formvar-covered nickel grids followed by overnight incubation with the α-EhNCX (1:20) antibody and later, by overnight incubation with the secondary antibody (1:60) conjugated to 15 nm gold particles (Ted Pella Inc.). Finally, sections were observed with a morgana 268 D Philips transmission electron microscope (FEI Company).

### Overexpression of the *EhCCX* gene

The full-length *Ehccx* gene was obtained by PCR using genomic DNA as template and using the conditions described above. Then, the amplicon was sequenced and cloned into the *BamHI* and *Kpnl* restriction sites of the *pExEhNeo* (*pNeo*) plasmid (Hamann et al., 1995), obtaining the *pNeo/Ehccx* construct.

For transfection, 3 × 10^5^ trophozoites were cultivated overnight at 37°C in a 5% CO_2_ environment; then, cells were washed with M199 medium (Sigma), resuspended in 1.8 ml of M199 medium supplemented with 15% of fetal bovine serum (FBS), and placed in 35-mm Petri dishes. Subsequently, a mix of 10 μg of plasmid (*pNeo* or *pNeo/Ehccx*) and 10 μg of Superfect (Qiagen) in 100 μl of M199 medium was added to the cultures and incubated for 4 h at 37°C in a 5% CO_2_ environment. Afterwards, cells were transferred to a tube containing 10 ml of TYI-S-33 pre-warmed medium and incubated 48 h at 37°C. Finally, transfected parasites were selected in the presence of 10 μg/ml of G-418 (Sigma-Aldrich). Overexpression was confirmed by qRT-PCR and Western blot assays.

For qRT-PCR, 100 ng of cDNA, 0.15 μM of each primer (forward, 5′-CACTGAAACACAAATCCCTTC-3′; reverse, 5′-CCAACTGAATTTCCCCAACA-3′) and the KAPA SYBR FAST PCR Master Mix (Kapa Biosystems) were used in a StepOne TM Real-Time PCR System (Applied Biosystem). As normalizer we used the gene encoding for the protein S2 of the 40S ribosomal subunit (primers: forward, 5′-ATTCGGAAATAGAAGAGGAGG-3′; reverse, 5′- CTATTCTTCCAAGCTTGGT-3′). Data from three independent cDNA preparations were analyzed using the 2^−ΔΔCT^ method.

Western blot assays and semi-quantitative comparisons were performed as described above to analyze the expression level of the EhCCX protein in transfected trophozoites. The protein level in trophozoites transfected with the empty vector (pNeo) was arbitrary taken as 1.

### Intracellular Ca^2+^ levels

The trophozoites (1 × 10^6^) were suspended in 1 ml of PBS and incubated for 30 min at 37°C in the dark with 4 μM of the Ca^2+^-sensitive dye Fluo-4 AM (Invitrogen). To remove the extracellular dye, the parasites were washed three times with PBS and resuspended in 1 ml of Ca^2+^/Mg^2+^ PBS (CaCl_2_ 0.1 mM, MgCl_2_ 1 mM). The fluorescence signal was detected by flow cytometry using the 520/40 filters in a BD FACSCalibur analyzer (BD Biosciences). To analyze the cytosolic calcium levels during the programmed cell death, H_2_O_2_ 1 mM was added to the samples and the fluorescence intensity was measured every 40 s.

### Cell death induced by hydrogen peroxide

The trophozoites (5 × 10^5^) were treated with H_2_O_2_ 1 mM in serum-free TYI medium and incubated at 37°C. Then, the cell viability was measured at different times by Trypan Blue exclusion.

### Erythrophagocytosis

Trophozoites suspended in serum-free TYI medium were incubated with fresh RBCs (1:100 ratio) at 37°C with slight agitation for 5, 10, and 15 min. Non-ingested erythrocytes were lysed by incubation with distilled water for 10 min at room temperature. Then, the samples were washed three times with PBS and parasites with internalized RBCs, were lysed with 1 ml of concentrated formic acid (J.T. Baker). Finally, phagocytosis was determined by measuring the hemoglobin released by the ingested erythrocytes (absorbance at 405 nm using a Beckman Coulter DU800 spectrophotometer).

### Cytopathic assays

Cytopathic assays, defined as the ability of live trophozoites to destroy cultured cells, were carried out as previously described (Rodríguez and Orozco, 1986). Briefly, MDCK cell monolayers (1 × 10^5^ cells) were incubated for 2 h at 37°C with 1 × 10^5^ trophozoites. Then, the trophozoites were eliminated by incubation at 4°C during 10 min, and the remaining mammalian cells were fixed with 2.5% (v/v) glutaraldehyde and stained with 1% (w/v) methylene blue. After exhaustive washes, the dye captured by cells was extracted with 0.1 M HCl and measured in a spectrophotometer (Beckman Coulter DU800) at 660 nm.

### Migration assays

Serum-starved (3 h) trophozoites (7.5 × 10^4^) were placed in the upper chamber of transwell inserts (5 μm pore size, 24 well, Costar) and 500 μl of adult bovine serum was added to the lower chamber. Then, trophozoites were incubated for 3 h at 37°C and migration was determined by counting the number of trophozoites at the lower chamber of the well.

### Statistical analysis

Values of all assays were expressed as the mean ± standard error of three independent experiments by duplicate. Statistical analyzes were carried out using the GraphPad Prism V5.01 software by two-way ANOVA and Student's *t*-test.

## Results

### *E. histolytica* has a calcium exchanger belonging to the CCX family

We search for putative calcium/cation exchangers in the Amoeba Genomics Resource (http://www.amoebaDb.org) using as probes the α1 and α2 domains of the different human calcium/cation exchangers (NCX, NCKX, or NCLX). By this analysis, the α1 domains of NCLX and NCX2, as well as the α2 domains of NCLX, NCKX1, and NCKX2 retrieved just one putative cation/calcium exchanger in *E. histolytica* (EHI_001770), *E. nuttali* (ENVI_134420), *E. dispar* (EDI_138590), and *E. invadens* (EIN_083040), whereas they retrieved two exchangers of *E. moshkovskii* (EMO_063580 and EMO_049830). The identity of the *E. histolytica* exchanger with the orthologous proteins of the other *Entamoeba* species ranged from 69 to 99%, whereas similarity varied from 83 to 99% (Table 1). On the other hand, the *E. histolytica* exchanger displayed 16–30% identity and 42–51% similarity with calcium/sodium exchangers from plants, mammals, amphibians, insects, and bacteria (Table 1).

**Table 1:** Comparison of the calcium/sodium exchanger of *E. histolytica* with orthologous proteins of different organisms.

**Organism**	**Accession number**	**Long (aa)**	**Identity (%)**	**Similarity (%)**	**E value**
*Entamoeba nutalli*	ENVI_134420	513	99	99	0.0
*Entamoeba dispar*	EDI_138590	510	95	97	0.0
*Entamoeba invadens*	EIN_083040	515	67	82	0.0
*Entamoeba moshkovskii*	EMO_063580	381	74	86	0.0
*Entamoeba moshkovskii*	EMO_049830	373	69	83	0.0
*Arabidopsis thaliana*	NP_197288.1	570	27	51	6e−51
*Aplysia californica*	XP_012946734.1	559	28	49	4e−49
*Eucalyptus grandis*	XP_010054949.1	604	30	53	2e−48
*Xenopus laevis*	NP_001128697.1	548	26	50	1e−46
*Homo sapiens*	NP_079235.2	584	24	46	9e−42
*Anopheles gambiae*	XP_308488.4	535	27	47	2e−41
*Homo sapiens*	NP_995322.1	500	20	43	1e−18
*Homo sapiens*	NP_066920.1	973	16	42	6e−06
*Methanococcus jannaschii*	3V5S_A	320	17	46	5e−05

**Figure 1:**
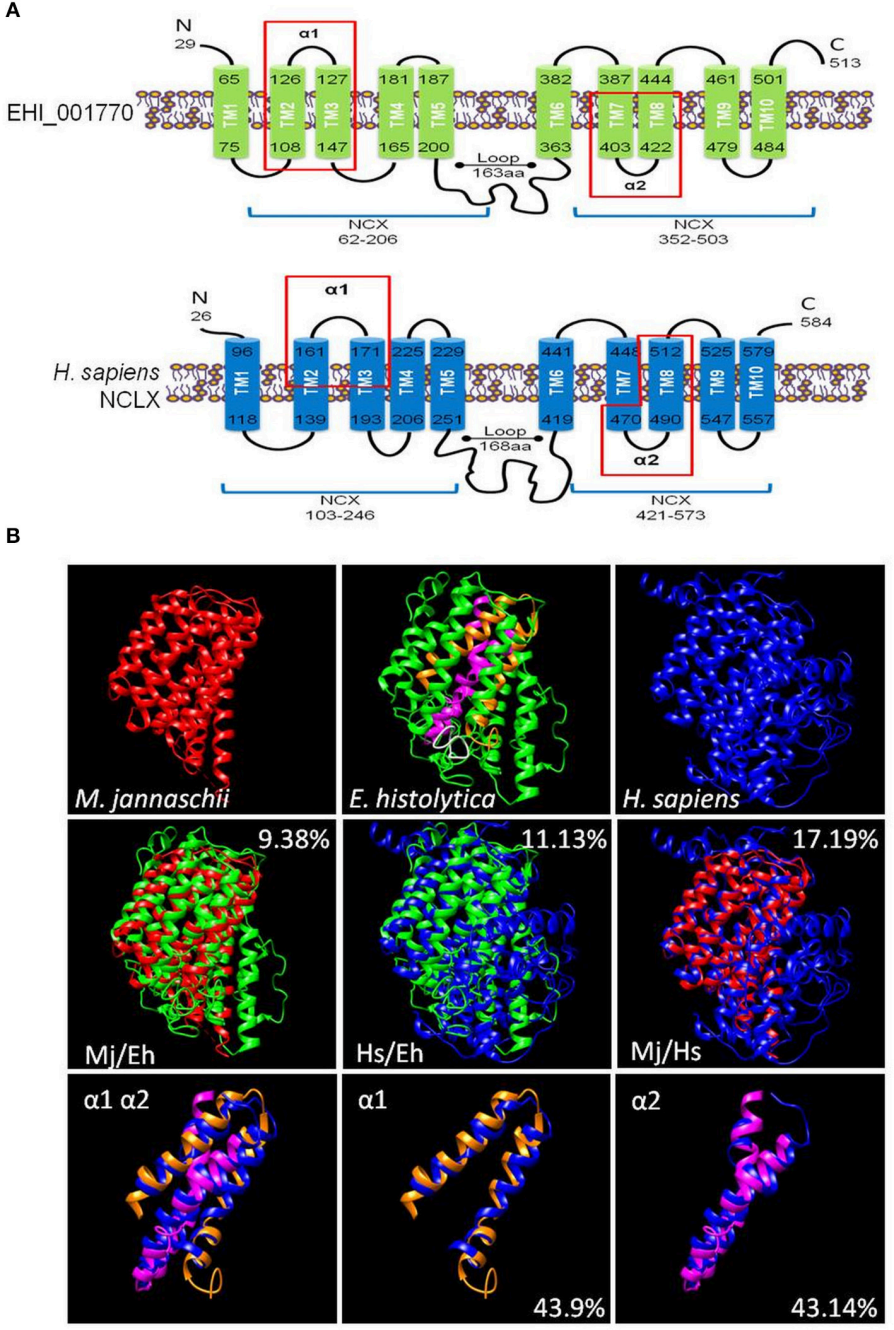
*In silico* characterization of a calcium/cation exchanger of *E. histolytica*. **(A)** Secondary structure models of the putative calcium/cation exchanger of *E. histolytica* and the human NCLX. The signal peptides were eliminated in the models. Numbers indicate the amino acid position. Bars represent the transmembrane regions (TM1 to TM10). The protein segments that correspond to the NCX domains are indicated by blue lines below the models. Red rectangles showed the protein segments corresponding to the α1 and α2 repeats. **(B)** The 3D models of the *E. histolytica* exchanger (green) and the human NCLX (blue) were predicted by the I-TASSER server using the crystallized sodium/calcium exchanger of *Methanococcus jannaschii* NCX (red) as a template. The white portion in the 3D model of the *E. histolytica* protein represents the peptide used to obtain specific antibodies against it, whereas orange and magenta segments correspond to the α1 and α2 repeats, respectively. The overlapping of the 3D models of the whole proteins as well as of the α1 and α2 domains are also shown. Numbers indicate the percentage of structural identity.

The putative calcium/cation exchanger of *E. histolytica* has 513 amino acid residues. It contains a signal peptide in positions 1 to 19 and its secondary structure model showed a similar organization to the human NCLX protein (NP_079235.2) (Figure 1A). Both proteins contain: (i) two sodium/calcium exchanger (NCX) domains (positions 62-206 and 352-503 in EHI_001770) that include the α1 and α2 repeats (positions 101-141 and 391-447 in EHI_001770); and (ii)10 putative transmembrane segments (positions 65-75, 108-126, 127-147, 165-181, 187-200, 363-382, 387-403, 422-444, 461-479, and 484-501 in EHI_001770) (Figure 1A). Then, we constructed a predicted 3D model of the putative *E. histolytica* exchanger using the crystallized structure of the sodium/calcium exchanger of *Methanococcus jannaschii* (MjNCX) as template. This 3D model confirmed the presence of 10 transmembrane segments in the amoebic protein, but it showed a modest structural identity with MjNCX (9.38%) and with the human NCLX (11.13%) (Figure 1B). However, the structural identity between human and bacteria exchangers was also moderate (17.19%) (Figure 1B). Nevertheless, the predicted structures of the α1 and α2 repeats of the *E. histolytica* protein showed a higher identity with those of NCLX (43.9 and 43.14%, respectively) (Figure 1B).

**Figure 2:**
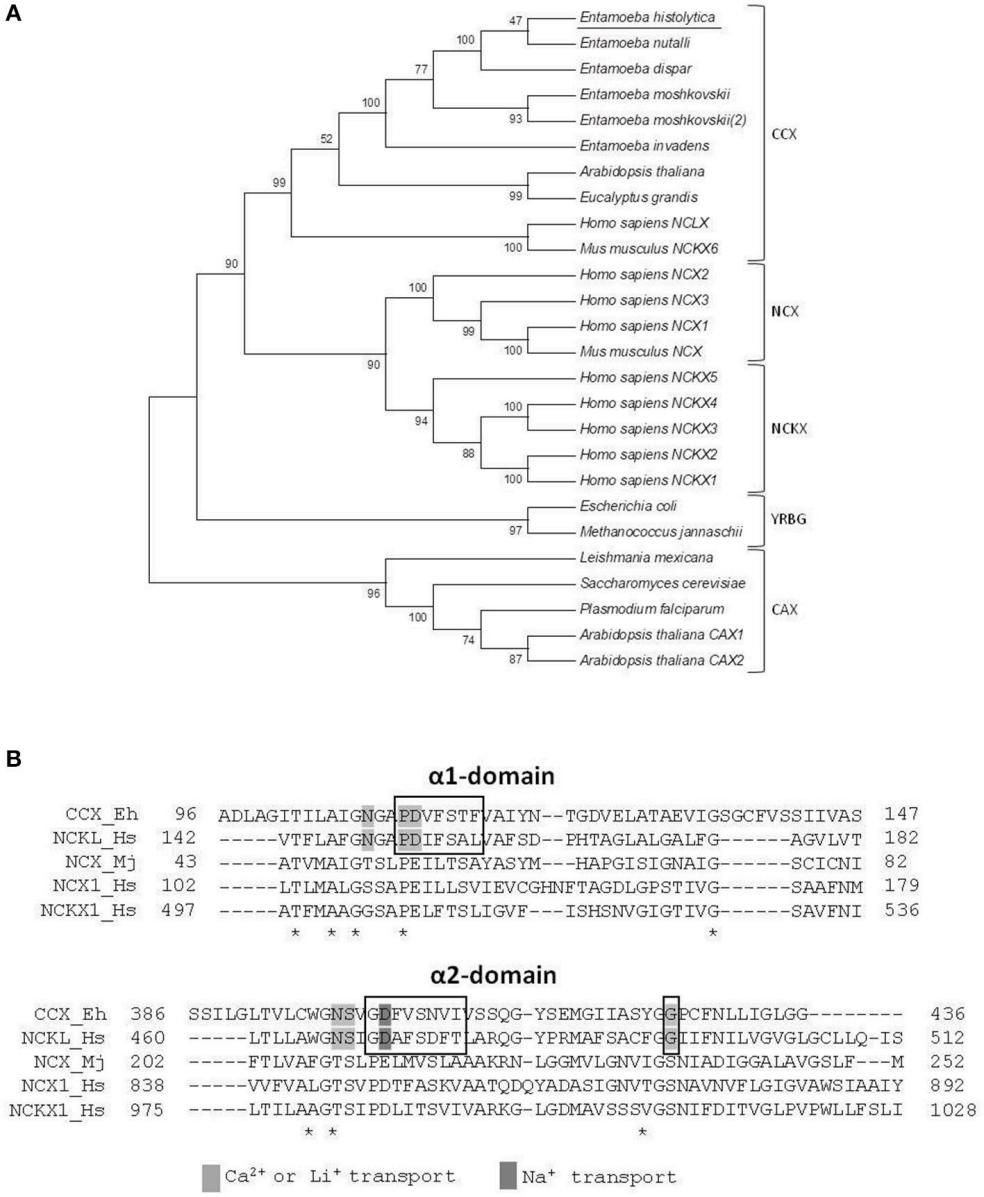
Phylogenetic analysis of the *E. histolytica* exchanger. **(A)** The predicted amino acid sequence of the *E. histolytica* exchanger was aligned by ClustalW with sequences of several calcium/cation exchangers from different organisms and data were submitted to phylogenetic analysis by UPGMA using the MEGA 5.05 software package. The protein sequences used for this analysis were: *E. histolytica* (accession number, EHI_001770); *E. nutalli* (ENVI_134420); *E. dispqr* (EDI_138590); *E. invadens* (EIN_083040); *E. moshcovskii* (EMO_063580 and EMO_049830); *Homo sapiens* NCXL (NP_079235.2), NCKX 1-5 (NP_004718.1, NP_065077.1, NP_065740.2, NP_705932.2, and NP_995322.1), and NCX1-3 (NP_066920.1, NP_055878.1, and NP_150287.1); *Mus musculus* NCKX6 (NP_573484.2) and NCX (AAB69167.1); *Arabidopsis thaliana* NCKX (NP_197288.1), and CAX1-2 (NP_181352.1 and NP_566452.1); *Eucalyptus grandis* CCX (XP_010054949.1); *Trichomonas vaginalis* CAX (XP_001582908.1); *Plasmodium falciparum* CAX (SCP05226.1); *Leishmania Mexicana* CAX (XP_003873397.1); *Saccharomyces cerevisiae* CVX1 (NP_010155.1); *Escherichia coli* NCX (WP_000922901.1); and *Methanococcus jannaschii* NCX (3V5S_A). Numbers at the branch nodes indicate the confidence percentages of the tree topology from bootstrap analysis of 1000 replicates. Proteins are grouped as cation/Ca^2+^ exchangers (CCX), Na^+^/Ca^2+^ exchangers (NCX), Na^+^/Ca^2+^ + K^+^ exchangers (NCKX), bacterial exchangers (YRGB), and H^+^/Ca^2+^ exchangers (CAX). **(B)** Amino acid sequence alignment of the α1 and α2 repeats of the *E. histolytica* exchanger with those of representative members of the different exchanger families. Asterisks indicate identical residues in all sequences. Boxes show the distinctive motives of the CCX family. Residues involved in calcium, sodium, or lithium transport are shadowed.

To determine the family of calcium exchangers to which the putative *E. histolytica* protein belongs, we compared its amino acid sequence with those of different members of the YRGB (bacterial exchangers), NCX (Ca^2+^/Na^+^ exchangers), NCKX (Ca^2+^/Na^+^ + K^+^ exchangers), CAX (Ca^2+^/H^+^ exchangers), and CCX (Ca^2+^/cation exchangers) families. Then, we constructed a phylogenetic tree, where the proteins of the different *Entamoeba* species were clustered into the clade comprising the CCX family, whereas the exchangers of other protozoa parasites were grouped in the clade of the CAX family (Figure 2A). Moreover, the *E. histolytica* exchanger contains the sequences GNG(A/T)PD and GNSIGD, corresponding to distinctive motives of the CCX family, which include the Ca^2+^/Na^+^ or Li^+^ exchangers (NCLX) (Sharma and O'Halloran, 2014) (Figure 2B). Concordantly, the *E. histolytica* protein shares all the amino acid residues that were identified as responsible of transporting Ca^2+^, Na^+^, or Li^+^ in the human NCLX (Roy et al., 2017) (Figure 2B); therefore, we named the *E. histolytica* exchanger as EhCCX.

### Expression and localization of EhCCX

**Figure 3:**
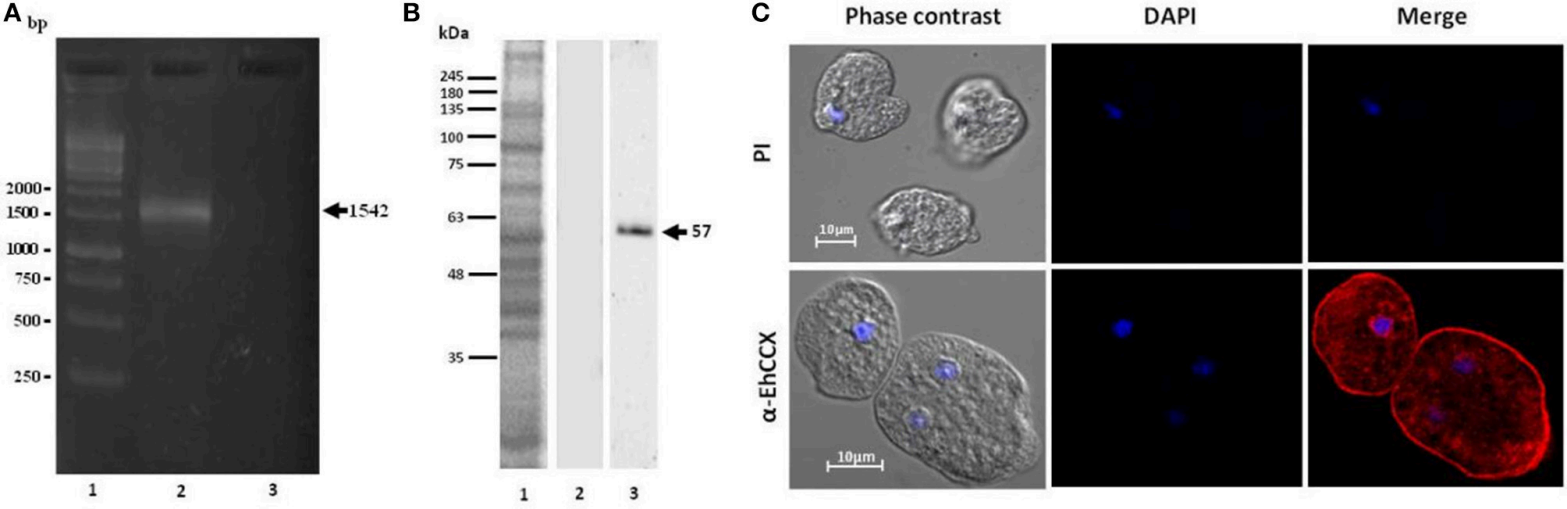
Expression of the *Ehccx* transcript and immunolocalization of the EhCCX protein. **(A)** Total RNA was obtained from *E. histolytica* trophozoites, cDNA was synthesized, and RT-PCR assays were performed using specific primers for *Ehccx*. Lane 1, 1 kb size markers. Lane 2, PCR assay using cDNA as template. Lane 3, PCR using RNA (omitting the cDNA synthesis) as template. **(B)** A specific peptide of EhCCX was synthesized and inoculated in rats to obtain antibodies against this exchanger. Then, to analyze their specificity, these antibodies were used in Western blot assays on total extracts of trophozoites. Lane 1, total proteins of *E. histolytica* trophozoites stained with Coomassie blue; lane 2, Western blot assay using the pre-immune serum; lane 3, Western blot assay using α-EhCCX; **(C)** *E. histolytica* trophozoites were fixed, permeabilized and incubated with rat antibodies against EhCCX. Subsequently, samples were incubated with Alexa 555-conjugated secondary antibodies. Finally, nuclei were stained with DAPI and samples were analyzed by confocal microscopy. PI, pre-immune serum.

**Figure 4:**
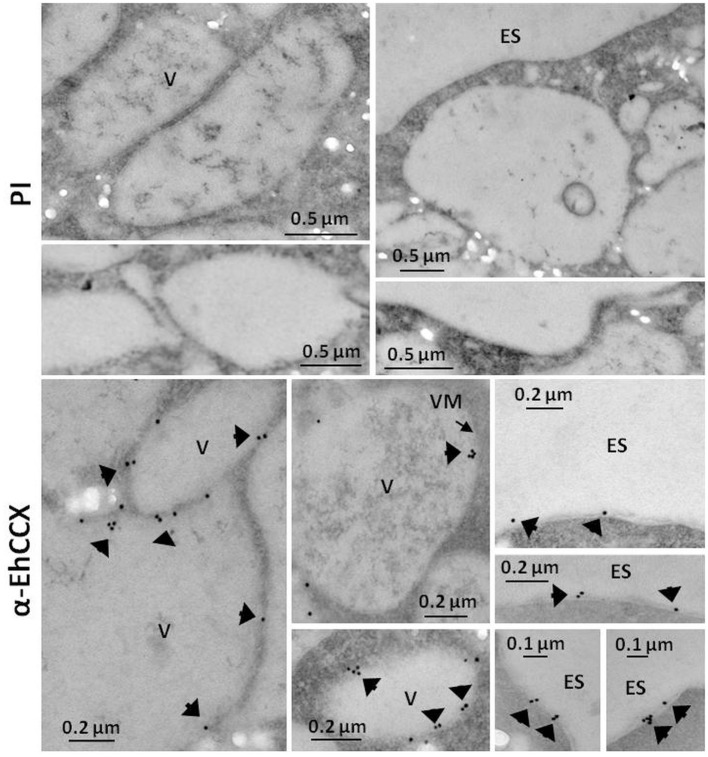
Immunoelectron microscopy. Thin sections (60 nm) of trophozoites were incubated with pre-immune serum (PI) or with antibodies against EhCCX (α-EhCCX) and then, with anti-rat IgGs conjugated to 15 nm gold particles. Afterwards, samples were analyzed by transmission electron microscopy. ES, extracellular space; V, vacuoles.

To investigate whether the transcript of the *Ehccx* gene is found in the trophozoites we performed RT-PCR assays. Results showed the amplification of a cDNA fragment with the expected molecular size (Figure 3A), indicating that the *Ehccx* gene is transcriptionally active under basal conditions. Next, with the purpose to immunodetect the EhCCX protein, we produced an antibody against a specific peptide located at position 216-229. Based on the 3D model, this peptide is into an exposed segment (Figure 2A). In Western blot assays, the antibody detected a single band of 56 kDa (Figure 3B), the expected molecular weight for EhCCX. By immunofluorescence assays, this protein was detected in the plasma membrane and in the membrane of numerous cytoplasmic vesicles (Figure 3C). Immunoelectron microscopy confirmed the presence of EhCCX in the plasma membrane and in the membrane of some cytoplasmic vesicles (Figure 4).

**Figure 5:**
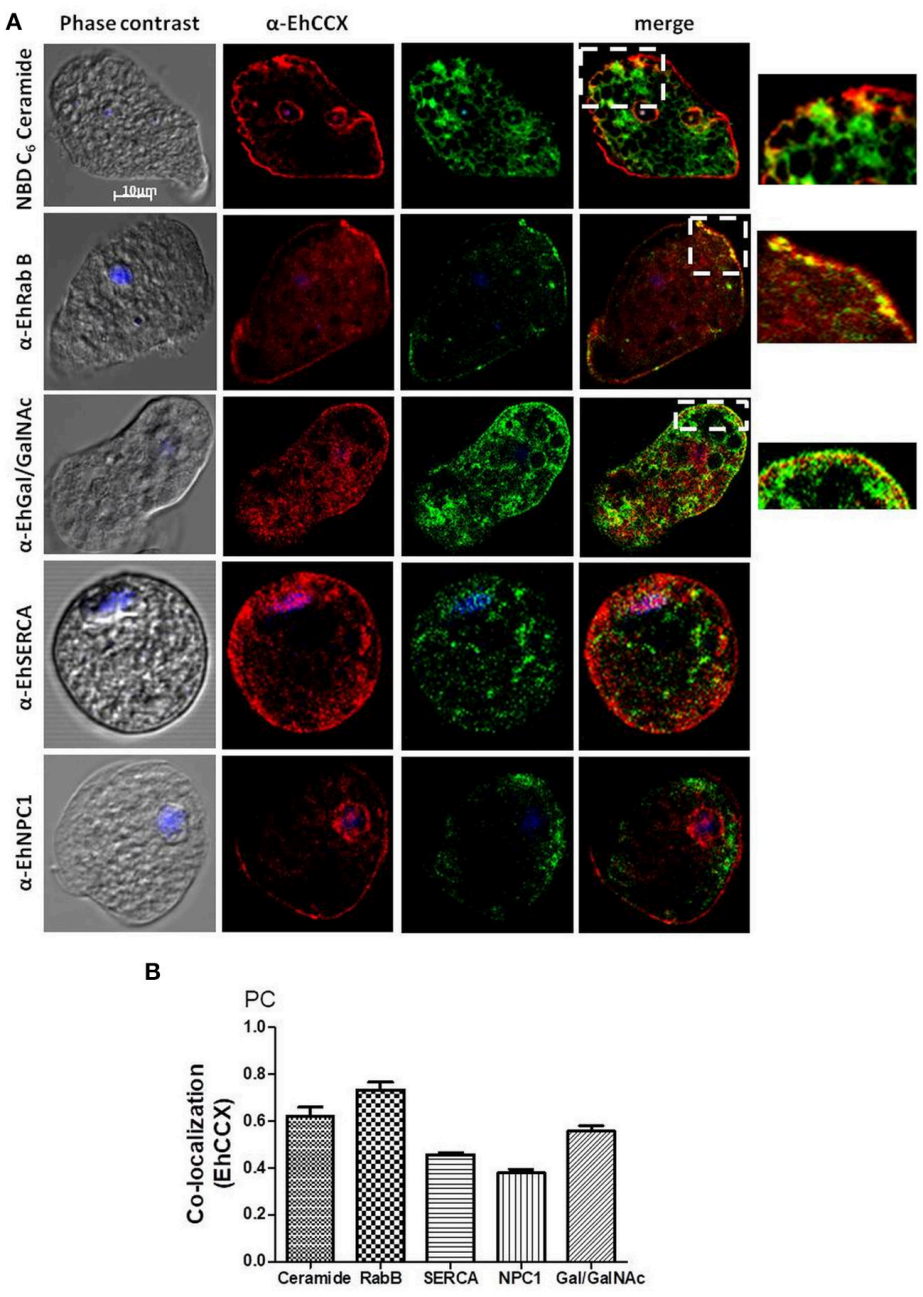
Co-localization assays. **(A)** *E. histolytica* trophozoites were fixed, permeabilized and incubated with rat antibodies against EhCCX and subsequently with rabbit antibodies against EhSERCA, EhRabB, or EhNPC1, or mouse antibodies against the Gal/GalNac lectin. Afterwards, samples were incubated with anti-rat IgGs labeled with Alexa 555, and with anti-rabbit IgGs labeled with Alexa 488 or anti-mouse IgGs labeled with FITC. Alternatively, after incubation with α-EhCCX and the anti-rat secondary antibody, trophozoites were incubated with NBD C_6_-ceramide. Finally, nuclei were stained with DAPI and samples were analyzed by confocal microscopy. On the right are shown magnifications of the squares marked in the merge images of co-localization with NBD C_6_-ceramide, EhRabB, and Gal/GalNac lectin. **(B)** Pearson coefficients of co-localization between EhCCX and the different markers.

To identify the possible cytoplasmic compartments in which EhCCX might be situated, we analyzed its co-localization with markers for endoplasmic reticulum (EhSERCA), Golgi apparatus (NBD C_6_-Ceramide), late endosomes (NPC-1), and endocytic vesicles (EhRabB). We also confirmed the presence of EhCCX in the plasma membrane by its co-localization with the Gal/GalNac lectin. In these assays, EhCCX showed some spots of co-localization with the Gal/GalNac lectin and EhRabB into or close to the plasma membrane (Figure 5A). This exchanger also showed co-localization with NBD C_6_-Ceramide in some vesicles close to the plasma membrane (Figure 5A), suggesting its presence in the trans-Golgi network. On the other hand, EhCCX did not display significant co-localization with EhSERCA, NPC-1 or cytoplasmic EhRabB (Figure 5A). The Pearson coefficient correlation confirmed a significant co-localization of EhCCX only with the Gal/GalNac lectin, EhRabB and NBD C_6_-Ceramide (Figure 5B). These results suggested that EhCCX in the plasma membrane could be involved in the extrusion of Ca^2+^ from cytosol, whereas its presence in in trans-Golgi could be due to its cytoplasmic transport. Alternatively, the posttranslational modifications needed for the maturation process of EhCCX could explain its presence in the Golgi apparatus. Nevertheless, we do not know the identity of most of the cytoplasmic vesicles that contain EhCCX nor its function in these structures.

**Figure 6:**
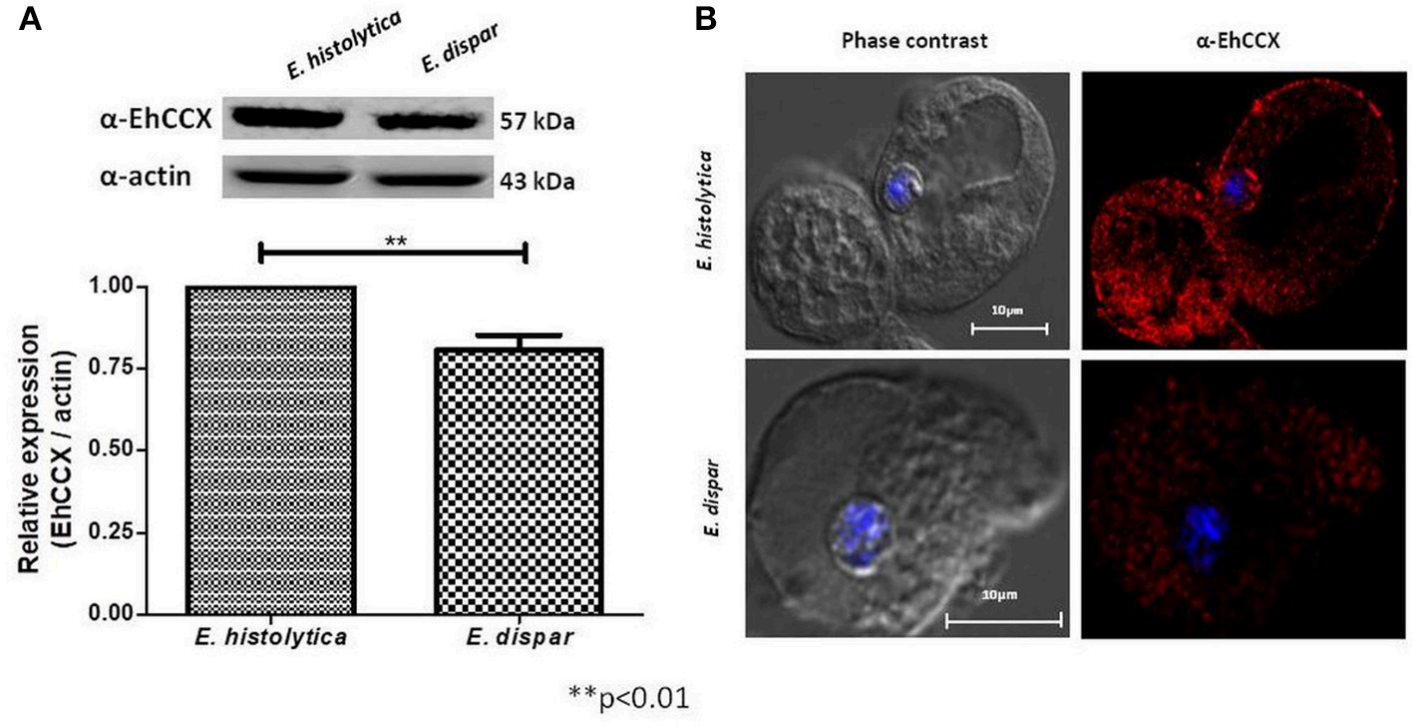
Expression and localization of CCX in *E. dispar*. **(A)** The comparison of the expression level of the CCX protein in *E. histolytica* (EhCCX) and *E. dispar* (EdCCX) was performed by Western blot using the α-EhCCX antibody. As an internal control, the membranes were probed with an α-actin antibody. The band detected by the α-EhCCX antibody was analyzed by densitometry and the data were normalized to the actin content. The relative expression in *E. histolytica* was taken as 1. Data are expressed as the mean ± standard error of three independent experiments. **(B)** *E. histolytica* and *E. dispar* trophozoites were fixed, permeabilized and incubated with α-EhCCX antibodies. Next, they were incubated with Alexa 555-conjugated secondary antibodies, nuclei were stained with DAPI, and samples were analyzed by confocal microscopy.

The non-pathogenic amoeba *E. dispar* has a lower erythrophagocytic capacity that the pathogenic *E. histolytica* (Talamás-Lara et al., 2014). To analyze whether EhCCX could participate in virulence, we compared the location and expression of the exchanger in *E. histolytica* (EhCCX) and *E. dispar* (EdCCX). Western blot assays showed that *E. histolytica* expresses a higher amount of the exchanger compared with *E. dispar* (Figure 6A). Immunofluorescence assays using the α-EhCCX antibody showed that the localization of the exchanger in the trophozoites of *E. histolytica* and *E. dispar* is similar, but the fluorescent signal was apparently lower in *E. dispar* (Figure 6B). The minor expression of the exchanger in *E. dispar* supports the hypothesis that EhCCX could participates in the virulence mechanism of *E. histolytica*.

### EhCCX is stress-inducible

**Figure 7:**
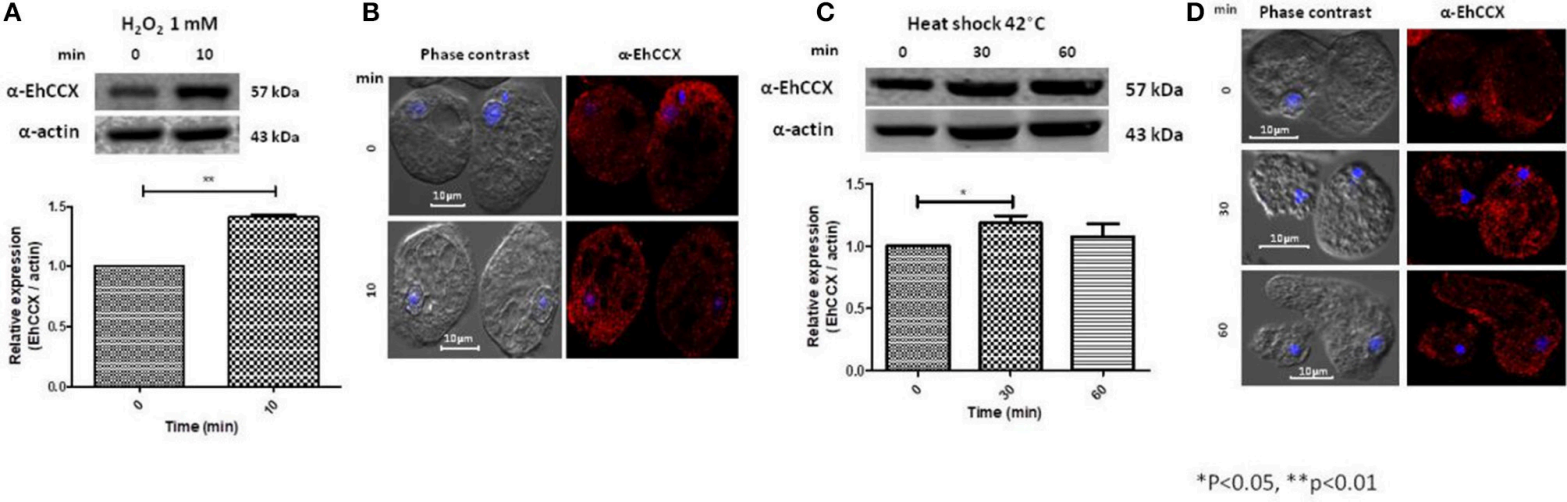
Expression and localization of EhCCX in trophozoites exposed to hydrogen peroxide and to heat shock. **(A,B)** *E. histolytica* trophozoites were incubated in the absence (0 min) or in the presence of hydrogen peroxide 1 mM during 10 min. **(C,D)** *E. histolytica* trophozoites were incubated at 37 °C (0 min) or at 42 °C during 30 and 60 min. **(A,C)** The expression of EhCCX was analyzed by Western blot. As an internal control, the membranes were probed with an α-actin antibody. The band detected by the α-EhCCX antibody was analyzed by densitometry and the data were normalized to the actin content. The relative expression in trophozoites at 0 min was taken as 1. Data are expressed as the mean ± standard error of three independent experiments. **(B,D)** Cells were fixed, permeabilized and incubated with α-EhCCX antibodies. Next, they were incubated with Alexa 555-conjugated secondary antibodies, nuclei were stained with DAPI, and samples were analyzed by confocal microscopy.

Calcium/cation exchangers are important in the restoration of the cytoplasmic concentration of Ca^2+^ after it has been elevated by different stimuli, including oxidative stress. Thus, we analyzed the expression level and localization of EhCCX in trophozoites exposed to H_2_O_2_ 1 mM during 10 min. Western blot assays showed that this exchanger slightly increased its expression (~0.4 times) under this condition (Figure 7A), suggesting that an augment of this protein could be needed to enhance the calcium extrusion trying to avoid the cellular damage. By immunofluorescence we observed a small accumulation of EhCCX in the plasma membrane and in some big vacuoles (Figure 7B).

We also analyzed the expression of EhCCX in trophozoites submitted to heat shock. After 30 min of incubation at 42°C, the expression of EhCCX showed a slight increase (about 0.2 times) that descend to approximately the basal levels after 60 min of heat shock (Figure 7C). We do not observe any change of the location of this exchanger under 30 and 60 min of incubation at 42°C (Figure 7D). Results suggest that EhCCX could has a minor participation or does not participate in the response to heat shock.

### Phagocytosis alters EhCCX localization

**Figure 8:**
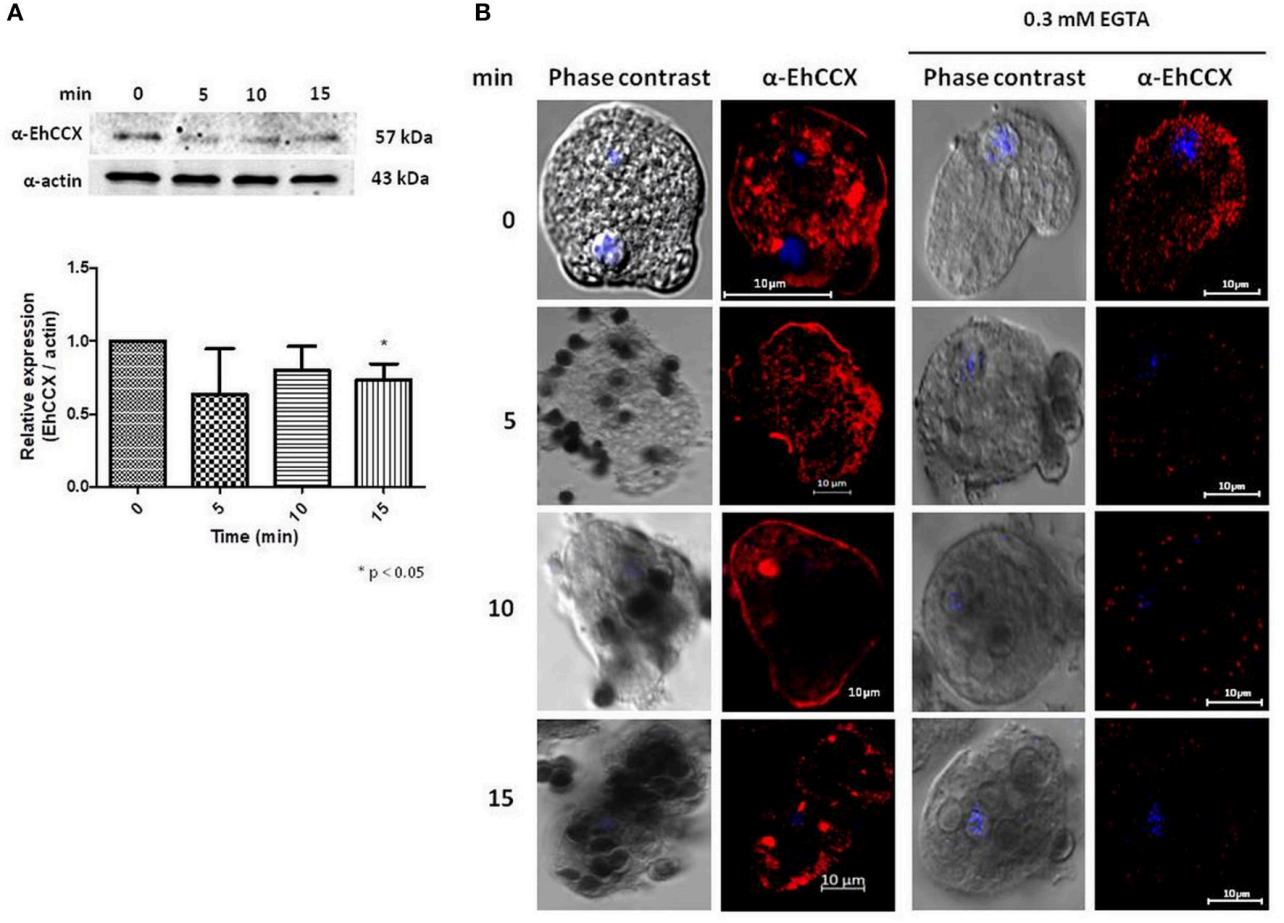
Expression and localization of EhCCX during phagocytosis. *E. histolytica* trophozoites were incubated at 37°C without erythrocytes (0 min) or in the presence of human erythrocytes during 5, 10, and 15 min. **(A)** The expression of EhCCX was analyzed by Western blot. As an internal control, the membranes were probed with an α-actin antibody. The band detected by the α-EhCCX antibody was analyzed by densitometry and the data were normalized to the actin content. The relative expression in trophozoites at 0 min was taken as 1. Data are expressed as the mean ± standard error of three independent experiments. **(B)** Cells were fixed, permeabilized and incubated with α-EhCCX antibodies. Next, they were incubated with Alexa 555-conjugated secondary antibodies, nuclei were stained with DAPI, and samples were analyzed by confocal microscopy. Images at the right correspond to assays performed in the presence of EGTA 0.3 mM.

It has been described that the accumulation of cytosolic calcium plays a major role in several cellular processes of *E. histolytica* trophozoites, including phagocytosis (Jain et al., 2008). Therefore, to investigate the possible role of EhCCX in this cellular event we performed Western blot assays on total extracts of trophozoites obtained at different times of erythrophagocytosis. No differences in the protein level were detected in these experiments (Figure 8A). However, we observed changes in its localization during the erythrophagocytosis. At 5 min, the localization of EhCCX was similar to the basal conditions; but at 10 min the exchanger was mainly detected in the plasma membrane, and after 15 min the presence of this protein diminished in the plasma membrane and it was concentrated in some cytoplasmic spots (Figure 8B). The accumulation of EhCCX in the plasma membrane at 10 min of phagocytosis suggested that this exchanger could participate in the signaling triggered by the interaction with the target cells and this signaling could be interrupted at 15 min by the internalization of EhCCX. In the presence of the calcium chelator EGTA, few erythrocytes were phagocyted by trophozoites, even after 30 min (Figure 8B), confirming that calcium flux is involved in this process. In this condition the movement of EhCCX to the plasma membrane was not observed, but it was concentrated in some small points in the cytoplasm (Figure 8B). These results suggest that the signaling pathway triggered by the presence of target cells promotes the recruitment of EhCCX in the amoeba surface.

### EhCCX overexpression modifies the cytoplasmic calcium level and PCD

**Figure 9:**
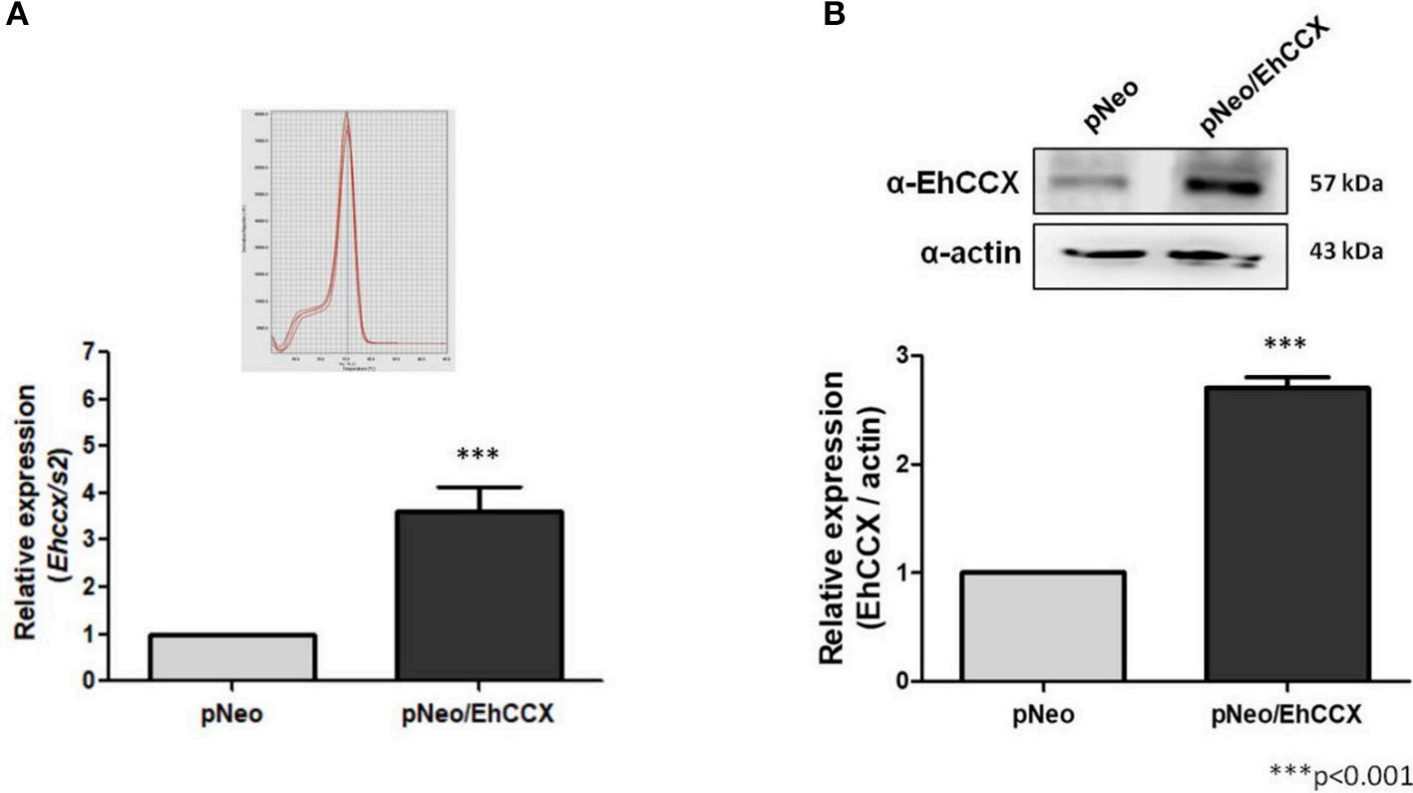
Overexpression of EhCCX. The *Ehccx* full-length gene was cloned into the pNeo vector and trophozoites were transfected with this construct (pNeo/EhCCX). Trophozoites transfected with the empty vector (pNeo) were used as controls. **(A)** RNA isolated from trophozoites was used for qRT-PCR assays. As gene normalizer we used the gene coding for the S2 protein of the 40S ribosome subunit. Relative expression was determined using the 2^−ΔΔCT^ method. Inset showed the melt curve that demonstrates the specificity of the *Ehccx* amplification. **(B)** Western bolt assays using the α-EhCCX antibody. As an internal control, the membranes were probed with an α-actin antibody. The band detected by the α-EhCCX antibody was analyzed by densitometry and the data were normalized to the actin content. The relative expression in pNeo trophozoites was taken as 1. Data are expressed as the mean ± standard error of three independent experiments.

To confirm the role of EhCCX in calcium movement and virulence we overexpressed this putative exchanger in trophozoites. Thus, we amplified the *Ehccx* gene by PCR using genomic DNA as a template and a high-fidelity DNA polymerase. The sequence of the amplicon showed two nucleotide changes with respect to the sequence EHI_001770 deposited in the genome database of *E. histolytica*: T to C in the position 220 and G to A in the position 328 (data not shown). These nucleotide substitutions modify the amino acid residues 74 (Ser to Pro) and 110 (Ala to Thr), which are into the α1 repeat (Supplementary Figure 1A); however, they did not produce significant alterations in the predicted 3D structure of this motif (Supplementary Figure 1B). In addition, although the residue in position 110 is into one of the distinctive sequences of the CCX members (Figure 2B), the amino acid change fits into the GNG(A/T)PD consensus sequence (Sharma and O'Halloran, 2014), and previous functional studies indicated that the residue situated in this place in the human NCLX does not participate in the ions transport (Roy et al., 2017). Thus, the amplicon was cloned into the *pExEhNeo* (*pNeo*) vector (Hamann et al., 1995) and trophozoites were transfected with the resultant construct (*pNeo/Ehccx*) to overexpress this exchanger. qRT-PCR assays showed that parasites transfected with the *pNeo/Ehccx* plasmid (pNeo/EhCCX trophozoites) overexpressed about 3.5 times the *Ehccx* transcript (Figure 9A). On the other hand, Western blot assays revealed that the EhCCX protein was overexpressed 2.8 times in pNeo/EhCCX trophozoites compared with the control (Figure 9B).

**Figure 10:**
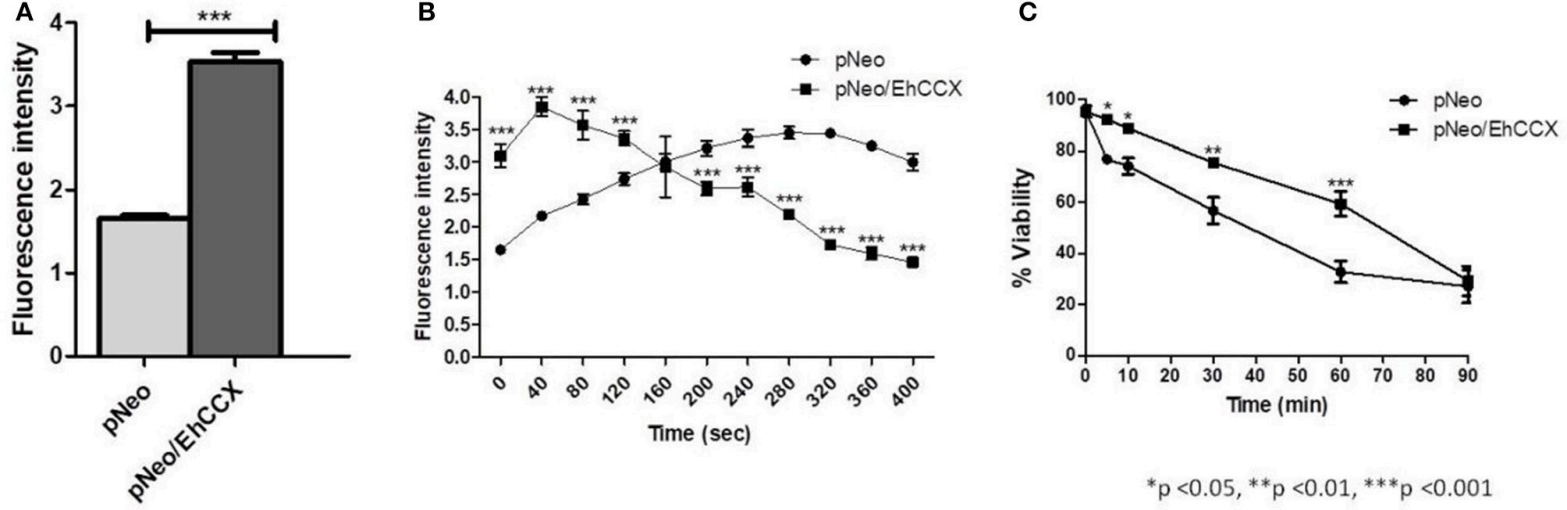
Effect of the EhCCX overexpression on the cytosolic calcium levels and PCD. **(A)** The cytosolic calcium level of pNeo and pNeo/EhCCX trophozoites in basal conditions was determined by using Fluo4 AM as described in Materials and Methods. **(B)** pNeo and pNeo/EhCCX trophozoites charged with Fluo4 AM were treated with hydrogen peroxide 1 mM and the fluorescence intensity was measured every 40 s. **(C)** The cell viability of trophozoites incubated with hydrogen peroxide 1 mM was determined by Trypan blue exclusion at 5, 10, 30, 60, and 90 min. Data are expressed as the mean ± standard error of three independent experiments performed by duplicate.

Then, to confirm that EhCCX participates in Ca^2+^ flux, we analyzed the cytosolic calcium levels in pNeo and pNeo/EhCCX cells using the Ca^2+^-sensitive dye Fluo-4 AM and flow cytometry. In these assays the fluorescence intensity in pNeo/EhCCX cells was about twice higher with respect to that showed by the pNeo trophozoites (Figure 10A). Then, to analyze the calcium flux in these trophozoites, we added H_2_O_2_ 1 mM and analyzed the fluorescence intensity every 40 s. In these assays, the signal gradually increased in pNeo cells (Figure 10B). In contrast, in pNeo/EhCCX trophozoites the florescence intensity augmented after 40 s of incubation with hydrogen peroxide, but subsequently the signal progressively diminished until at 400 s the fluorescence intensity was similar to that of pNeo cells under basal conditions (Figure 10B). These results confirm the role of EhCCX in Ca^2+^ transport, because the higher expression of the exchanger produced a greater influx of calcium under basal conditions and improve the extrusion of the cytosolic calcium during the programmed cell death (PCD) induced by H_2_O_2_.

In concordance with the augmented extrusion of calcium during the PCD-induction, after 30 and 60 min of incubation with H_2_O_2_, the cell death of pNeo/EhCCX was significantly lower than pNeo (Figure 10C); however, we did not observe differences in the cell death after 90 min of treatment (Figure 10C). These results suggest that the higher extrusion of cytosolic calcium driven by the overexpressed exchanger retarded the PCD of trophozoites.

### EhCCX overexpression is increasing the *in vitro* virulence of *E. histolytica*

**Figure 11:**
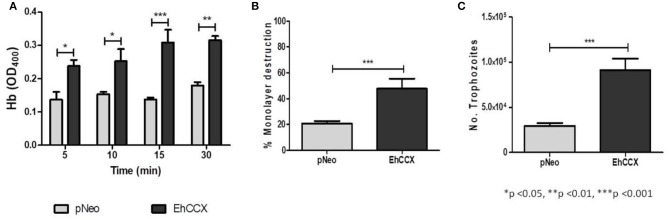
Effect of the EhCCX overexpression on the *in vitro* virulence. **(A)** pNeo and pNeo/EhCCX trophozoites were incubated with human erythrocytes at 37°C during 5, 10, 15, and 30 min. Then, non-ingested erythrocytes were lysed by incubation by 10 min with distilled water. After several washes with PBS, trophozoites were lysed with formic acid; and the hemoglobin released from the erythrocytes ingested was determined by absorbance at 405 nm. **(B)** MDCK cell monolayers were incubated with pNeo or pNeo/EhCCX trophozoites during 2 h at 37°C, and the monolayer destruction was evaluated as described in Material and Methods. **(C)** Serum-starved trophozoites were placed in the upper chamber of transwell inserts and 500 μl of adult bovine serum was added to the lower chamber. Then, trophozoites were incubated for 3 h at 37°C and migration was determined by counting the number of trophozoites at the lower chamber of the well. Data are expressed as the mean ± standard error of three independent experiments performed by duplicate.

We analyzed the erythrophagocytosis, cytopathic activity and migration of pNeo/EhCCX to corroborate that EhCCX is also involved in the *in vitro* virulence of *E. histolytica*. Results showed that pNeo/EhCCX cells displayed a significant increase in erythrophagocytosis, compared with pNeo (Figure 11A). EhCCX-overexpressing trophozoites also enhanced more than twice their ability to destroy mammalian cells (Figure 11B), and remarkably, migration of trophozoites augmented almost four times compared to pNeo (Figure 11C). All these results support the hypothesis that Ca^2+^ flux mediated by EhCCX participates in the *in vitro* virulence of *E. histolytica*.

## Discussion

The Ca^2+^ extrusion from the cytosol is carried out mainly by Ca^2+^-ATPases of the PMCA family and calcium/cations exchangers, therefore, these proteins play a critical role in Ca^2+^ homeostasis (Berridge et al., 2003). Calcium/cation exchangers transport Ca^2+^ against its electrochemical gradient coupled to the exchange of different cations, such as H^+^ (YRGB and CAX), Na^+^ (NCX), Na^+^ + K^+^ (NCKX), and Na^+^ or Li^+^ (CCX) (Lytton, 2007; Emery et al., 2012). All these exchangers share structural similarities, including two α-repeats involved in the ion binding/transport events (Giladi et al., 2016). The differences in the ion-coordinating residues among the members of the Ca^2+^/cation superfamily are responsible of the divergences in ion selectivity (Refaeli et al., 2016).

Ca^2+^ homeostasis is involved in the host invasion by different protozoa parasites (Ravdin et al., 1985; Lu et al., 1997; Vieira and Moreno, 2000; Lovett and Sibley, 2003; Moreno et al., 2007). It has been described the presence of PMCAs in most of these microorganisms (Moreno and Docampo, 2003), however, until now there are not reports about the existence of Ca^2+^/Na^+^ exchangers for any parasite protozoa. A genomic analysis in Apicomplexans revealed that *Toxoplasma gondii, Cryptosporidium* spp., and *Plasmodium* spp. contain orthologs of Ca^2+^/H^+^ exchangers (CAX) found in plants and yeast, but not in animals. Conversely, Ca^2+^/Na^+^ exchangers, which are common in animals, are not found in these parasites (Nagamune and Sibley, 2006). In *Plasmodium berghei*, the CAX protein participates in ookinete development and differentiation (Guttery et al., 2013). On the other hand, biochemical evidence suggests that trypanosomatids also have a Ca^2+^/H^+^ antiporter (Verseci et al., 1994), which was proposed to be involved in Ca^2+^ release by the increase of sodium mediated by a Na^+^/H^+^ antiporter (Versesi and Docampo, 1996). Concordantly, by a *in silico* analysis we found a gene coding for a putative Ca^2+^/H^+^ exchanger in *Leishmania mexiana*. We also identify a gene coding for a putative Ca^2+^/H^+^ exchanger in *Trichomonas vaginalis*, but we did not discover Ca^2+^/Na^+^ exchangers in any parasite protozoa different to *Entamoeba* spp.

The *E. histolytica* exchanger showed a higher similarity with the CCX family and contains the same amino acid residues involved in the transport of calcium, sodium, and lithium of human NCLX. It is known that the intracellular concentration of Li^+^ is very low (0.6–0.8 mM); thus, the Li^+^ transport by NCLX members just could be related to some functional properties. For instance, Ca^2+^ transport by NCX is driven by a steep Na^+^ gradient and a moderate (~-70 mV) membrane potential, whereas Ca^2+^ efflux by NCLX is primarily driven by a much steeper (~−200 mV) membrane potential (Roy et al., 2017).

Based on the disulfide cross-linking data, a model for Ca^2+^/Na^+^ exchangers was proposed (Nicoll et al., 1999). In this model, the transmembrane segments 5 and 6 (TMS5 and TMS6) are separated by a large (~500 amino acids) f-loop that faces the cytosolic side. This loop is responsible for the regulation of the ions transport activity by the binding of several cytoplasmic messengers, protons, NO, PIP_2_, phosphoarginine, phosphocreatinine, ATP, and endogenous inhibitors (Khananshvili, 2013). Interestingly, NCLX members have a very short f-loop with no regulatory domains on it (Khananshvili, 2013). Similarly, EhCCX has a small f-loop (163 aa), suggesting that this protein, as NCLX members, has no controlling domains for “secondary” regulation.

The human NCLX is found in the inner membrane of the mitochondria (Palty et al., 2010); however, it was also detected in the plasma membrane of ventricular myocytes and pancreatic β-cells (Cai and Lytton, 2004; Han et al., 2015). Indeed, it has been reported that in pancreatic β-cells the NCLX of plasma membrane is involved in the calcium flux that contributes to the vesicle recruitment for sustained exocytosis in response to repetitive depolarization (Han et al., 2015). Likewise, the EhCCX located in the surface of trophozoites may participates in the calcium flux through the plasma membrane. This hypothesis is supported by the increase of its expression in trophozoites exposed to H_2_0_2_ and by the changes in the cytosolic calcium levels in EhCCX-overexpressing trophozoites during the incubation with H_2_0_2._

On the other hand, EhCCX seems to be involved in the amoeba virulence. This hypothesis is supported by: (i) the expression level of this protein is higher in the pathogenic *E. histolytica* than in the non-pathogenic *E. dispar*; and (ii) the overexpression of EhCCX augmented the *in vitro* virulence properties of trophozoites, such as phagocytosis, destruction of mammalian cell monolayers and migration. Similarly, the overexpression of NCX proteins enhances the heart rate mediated by beta-adrenergic (Kaese et al., 2017), and the relaxation of gastric fundus (Fujimoto et al., 2016). We are hypothesizing that, as calcium/cation exchangers can reverse the ions transport under certain conditions (Harper and Sage, 2016), the different stimuli that are involved in the *E. histolytica* virulence could increase the Ca^2+^ influx via EhCCX, extending the period of high Ca^2+^ levels in cytoplasm, maintaining the response to the signal. Such mechanism of Ca^2+^ influx via NCX has also been proposed to explain the enhanced contraction displayed by the urinary bladder smooth muscles of transgenic mice overexpressing NCX1.3 (Yamamura et al., 2013).

In conclusion, in this work we identified a calcium/cation exchanger of *E. histolytica* (EhCCX). The expression of this exchanger, belonging to the CCX family, is higher in pathogenic than in non-pathogenic amoeba. Moreover, the cellular localization of EhCCX changed during phagocytosis and its overexpression increased the *in vitro* virulence and retarded the PCD induced by H_2_O_2_. These results suggest that Ca^2+^ flux through EhCCX participates in the PCD and the *in vitro* virulence of *E. histolytica*.

## Author contributions

MV-S conceived and carried out experiments, analyzed data, and drafted the manuscript. JB performed experiments and analyzed data. EO and MH participated in the design of the study and analyzed data. GG-R carried out the transfection of trophozoites and participates in their characterization. AS-C realized the measurements of cytosolic calcium levels. BC-M performed the immunoelectron microscopy. MR conceived and designed the study, analyzed data and drafted the manuscript.

### Conflict of interest statement

The authors declare that the research was conducted in the absence of any commercial or financial relationships that could be construed as a potential conflict of interest.

## Abbreviations

CCX: calcium/cation exchanger
EhCCX: calcium/cation exchanger of *E. histolytica*
EdCCX: calcium/cation exchanger of *E. dispar*
EhNPC1: Niemann-Pick type C protein 1 of *E. histolytica*
EhRabB: RabB protein of *E. histolytica*
EhSERCA: sarco/endoplasmic reticulum Ca^2+^-ATPase of *E. histolytica*
Gal/GalNac lectin: lectin of *E. histolytica* inhibited by galactose and N-acetyl-D-galactosamine
NCLX: sodium/calcium/lithium exchanger
NCX: sodium/calcium exchanger
MjNCX: sodium/calcium exchanger of *Methanococcus jannaschii*
PMCA: plama membrane Ca^2+^-ATPase
PCD: programmed cell death.
